# Lead exposure induces nitrative stress and disrupts ribbon synapses in the cochlea

**DOI:** 10.3389/fnmol.2022.934630

**Published:** 2022-07-27

**Authors:** Rita Rosati, Johnna A. Birbeck, Judy Westrick, Samson Jamesdaniel

**Affiliations:** ^1^Institute of Environmental Health Sciences, Wayne State University, Detroit, MI, United States; ^2^Lumigen Instrument Center, Wayne State University, Detroit, MI, United States; ^3^Department of Family Medicine and Public Health Sciences, Wayne State University, Detroit, MI, United States

**Keywords:** lead exposure, cochlear synaptopathy, nitrative stress, ototoxicity, hidden hearing loss

## Abstract

Environmental exposure to heavy metal lead, a public health hazard in many post-industrial cities, causes hearing impairment upon long-term exposure. Lead-induced cochlear and vestibular dysfunction is well-documented in animal models. Although short-term exposure to lead at concentrations relevant to environmental settings does not cause significant shifts in hearing thresholds in adults, moderate- to low-level lead exposures induce neuronal damage and synaptic dysfunction. We reported that lead exposure induces oxidative stress in the mouse cochlea. However, lead-induced nitrative stress and potential damage to cochlear ribbon synapses are yet to be fully understood. Therefore, this study has evaluated cochlear synaptopathy and nitrative stress in young-adult mice exposed to 2 mM lead acetate for 28 days. Inductively coupled plasma mass spectrometry (ICP-MS) analysis indicated that this exposure significantly increased the blood lead levels. Assessment of hair cell loss by immunohistochemistry analysis and outer hair cell (OHC) activity by recording distortion product otoacoustic emissions (DPOAEs) indicated that the structure and function of the hair cells were not affected by lead exposure. However, this exposure significantly decreased the expression of C-terminal-binding protein-2 (CtBP2) and GluA2, pre- and post-synaptic protein markers in the inner hair cell synapses, particularly in the basal turn of the organ of Corti, suggesting lead-induced disruption of ribbon synapses. In addition, lead exposure significantly increased the nitrotyrosine levels in spiral ganglion cells, suggesting lead-induced nitrative stress in the cochlea. Collectively, these findings suggest that lead exposure even at levels that do not affect the OHCs induces cochlear nitrative stress and causes cochlear synaptopathy.

## Introduction

Exposure to lead, a ubiquitous and non-biodegradable environmental toxin (Flora et al., 2012), is a major public health concern. The World Health Organization has reported that lead exposure was associated with 1.06 million deaths worldwide in 2017 and accounted for 63.2% of global developmental intellectual disabilities in 2016. Small amounts of lead are found in the earth’s crust (Acharya, 2013) and high concentrations are found in older homes containing lead-based paints, ceramics, pipes and plumbing materials, batteries, ammunition, and cosmetics. Lead is absorbed by the human body through inhalation, ingestion, and dermal contact (Wani et al., 2015) and causes deleterious effects on cardiovascular, renal, skeletal, and nervous systems (Mitra et al., 2017). As a calcium ion substitute, lead can pass through the blood–brain barrier and accumulate in the nervous tissue, which results in the generation of reactive oxygen species (ROS), ultimately interfering with cell signaling and neurotransmission (Sharma et al., 2015). In addition to the well-known neurotoxic effects (Lee et al., 2019; Yang et al., 2019; Zhou et al., 2020), hearing impairment is an important adverse health outcome associated with long-term exposure to lead (Shargorodsky et al., 2011; Choi et al., 2012; Ghiasvand et al., 2016; Rosati and Jamesdaniel, 2020).

Lead exposure disrupts the structure and the function of the auditory system. A blood lead level of ≥ 2 μg/dl, which is below the action level (5 μg/dl) recommended by the Centers for Disease Control and Prevention (Rhoads et al., 2012), and bone lead levels of 15 μg/g (in the tibia) were associated with increased odds ratio of hearing loss (Park et al., 2010; Shargorodsky et al., 2011; Ghiasvand et al., 2016). Exposure of mice to even low levels of lead during the early developmental stage decreased the expression of voltage-dependent anion channel proteins and also disrupted the monoaminergic system in the auditory brainstem (Fortune and Lurie, 2009; Prins et al., 2010). Consistent with the observation in other tissues, where increased generation of ROS was detected (Ercal et al., 2001; Vaziri and Khan, 2007; Muthusamy et al., 2016), lead exposure induced oxidative stress in mouse cochlea (Jamesdaniel et al., 2018). Furthermore, animal studies have shown that exposure to lead induced degeneration of cochlear sensory receptor cells, disrupted cochlear blood-labyrinth barrier, affected auditory nerve conduction velocity, and caused vestibular dysfunction (Yamamura et al., 1989; Lasky et al., 1995; Jones et al., 2008; Liu et al., 2013; Klimpel et al., 2017). Despite such compelling evidence, the ototoxic potential of lead is underestimated because short-term exposure to lead at concentrations relevant to environmental settings does not cause significant shifts in hearing thresholds in adults (Carlson and Neitzel, 2018). However, such exposure can cause hidden hearing loss because lead is a potent neurotoxicant that induces synaptic dysfunction (Yang et al., 2019).

Cochlear synaptic transmission is a critical determinant of the quality of auditory perception. It is affected primarily in age-related, noise-induced, and drug-induced (e.g., aminoglycosides) auditory dysfunction long before significant hearing loss is detected (Liberman and Kujawa, 2017). The main target in these otopathologies is the ribbon synapses in the inner hair cells. Even moderate-level ototoxic exposures that do not alter hearing thresholds can damage the ribbon synapses in the inner hair cells, thereby affecting auditory perception (Fernandez et al., 2020). This silencing of subsets of cochlear nerve fibers can contribute to difficulty in hearing speech-in-noise, hyperacusis, and tinnitus, which are among the common hearing disorders caused by ototoxic insults. As the hair cell population is likely to be intact even when there is widespread cochlear synaptopathy, audiograms might not reveal the underlying otopathology. Although the neurotoxic properties of lead imply that environmental exposure can disrupt the cochlear ribbon synapses, lead-induced cochlear synaptopathy has not been studied. The synaptic proteins, which are pivotal for transduction of auditory signals, are highly susceptible to nitration (Bradley and Steinert, 2016). As nitrated proteins are susceptible to degradation, lead-induced cochlear nitrative stress can compromise the transmission and processing of auditory signals. Although we reported that lead exposure induced oxidative stress in the cochlea (Jamesdaniel et al., 2018), lead-induced cochlear nitrative stress, i.e., a sequela of oxidative stress, is yet to be fully understood.

The hypothesis of this study is that environmental exposure to lead at levels that do not affect hair cell viability induces cochlear nitrative stress and causes synaptopathy. To test this hypothesis, we quantified lead-induced cochlear nitrative stress by immunohistochemistry analysis of protein nitration in spiral ganglion cells and evaluated lead-induced disruption of cochlear ribbon synapses by immunohistochemistry analysis of pre- and post-synaptic biomarkers in a mouse model.

## Materials and methods

### Animals

**FIGURE 1:**
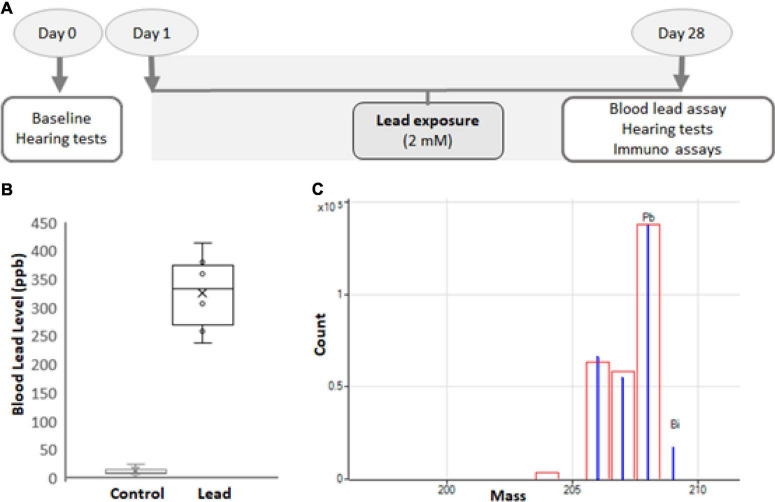
Experimental design. **(A)** Five-week-old male C57BL/6 mice were exposed to 2 mM lead acetate in water for 28 days. Hearing thresholds, hair cell loss, ribbon synapse disruption, and nitrotyrosine levels in spiral ganglions were assessed for 1 day after the last exposure. Lead load was analyzed by assessing blood lead levels. **(B)** The blood lead levels in mice that consumed water containing 2 mM lead acetate for 28 days were 326 ± 70 ppb. The results are expressed as mean ± standard deviation, *n* = 6. **(C)** Blood lead levels were analyzed using inductively coupled plasma mass spectrometry (ICP-MS). The mass spectrum (blue) of Pb and Bi (internal) standards used in the ICP-MS analysis is illustrated. The red box indicates the isotopic template.

C57BL6/J mice were purchased from Jackson Laboratories (Bar Harbor, ME, United States) and young-adult male mice (5-week-old) were used in this study. Although the strain C57BL6/J harbors mutations in Cdh23, these mice are considered good models for studying vestibular and cochlear ototoxicity, particularly before the onset of age-related hearing loss (Kane et al., 2012; Jamesdaniel et al., 2018). All animals were allowed to acclimatize for 5 days in a temperature-controlled room with a 12-h light/dark cycle and were housed in the Laboratory Animal Facility of Wayne State University. They had free access to food and water and the ambient noise in the room was maintained below 50 dB sound pressure level (SPL). All animals were handled and treated according to the guidelines established by the National Institutes of Health and every effort was made to minimize pain and discomfort. The experimental protocol (Figure 1A) was reviewed and approved by the Institutional Animal Care and Use Committee (#16-01-038).

### Lead exposure

The mice were randomly divided into 2 groups and baseline hearing tests were conducted in 5-week-old mice. Only animals with good baseline hearing were used in this study. The mice in the control group were given plain drinking water, while those in the experimental group were exposed to 2 mM lead acetate (Cat # 316512, Sigma-Aldrich, St. Louis, MO, United States) through drinking water for 28 days (Figure 1A; Jamesdaniel et al., 2018). The weight, appearance, and behavior of the animals were monitored routinely, and the animals were provided free access to standard mouse chow. To ensure all animals have adequate and similar exposure, three animals were housed in one cage.

### Distortion product otoacoustic emissions

Otoacoustic emissions were measured in a soundproof chamber (model AB-4230, ECKEL, Morrisburg, ON, United States). The animals were anesthetized with isoflurane (4% induction, 1.5% maintenance with 1.5 L/min O_2_) for measuring the distortion product otoacoustic emissions (DPOAEs). Two primary tones, f1 and f2 at an f2/f1 ratio of 1:2, were used for eliciting the DPOAEs by holding L2–L1 + 10 dB, for L1 levels from 80- to 20-dB SPL in 10-dB increments. The stimulus was generated by using the Tucker-Davis Technology (TDT) RZ6 system, and f1 and f2 were delivered by using multifield magnetic speakers (TDT, Alachua, FL, United States). Frequency f2 varied from 8 to 32 kHz. ER10B_ probe microphone (Etymotic Research, Inc., Elk Grove Village, IL, United States) along with hardware and software from TDTs was used to measure SPLs at the cubic difference frequency (2f1–f2). Distortion product data were collected every 20.971 ms and averaged 512 times. A 100-kHz band surrounding 2f1–f2 was used to measure the noise floor.

### Blood and cochleae collection

All animals were euthanized by CO_2_ inspiration. After ensuring that there is no response to a toe pinch, blood was collected by cardiac puncture (∼100–150 μl) and stored in 1.5 ml Eppendorf tubes containing 10 μl of 0.5 M ethylenediaminetetraacetic acid (EDTA). Then, the mice were sacrificed by cervical dislocation, decapitated, temporal bone was removed, and the cochlea was dissected out quickly in ice-cold phosphate-buffered saline (PBS). Then, the round and oval windows were opened and immediately perfused with 4% paraformaldehyde in PBS at pH 7.4 by slow injection *via* the round window. The perfused cochlear tissue was fixed overnight in the same solution at room temperature.

### Inductively coupled plasma mass spectrometry analysis

A 75-μl sample of blood was diluted with an equal volume of 0.1% Trition X-100. Then, 300 μl of 2% nitric acid was used for further dilution and the samples were incubated for 1–2 h. Samples were centrifuged and 2% nitric acid was used to dilute the samples again for a final 50-fold dilution. A standard curve was generated using standard concentrations of lead ranging from 0.05 to 200 ppb. Lead and bismuth (internal) standards (Figure 1C) were purchased from Inorganic Ventures (Christiansburg, VA, United States), and the analysis was performed on an Agilent 7700X Series inductively coupled plasma mass spectrometry (ICP-MS).

### Cochlear microdissection

All cochleae were decalcified in 100 mM EDTA solution for 3 days. Then, the organ of Corti was dissected out of the cochlea under a dissecting microscope in a 0.01-mM PBS solution. The basilar membrane from the organ of Corti was further microdissected into 3 major sections, representing the base, middle, and apical turns of the cochlea (Rosati et al., 2021). These sections were used for immunohistochemistry analysis.

### Immunohistochemistry

The basal, middle, and apical turns from the cochlear basilar membrane were permeabilized and blocked in a solution containing PBS, 1% v/v Triton X-100, 2% w/v bovine serum albumin, and 10% v/v goat serum (Cat # S26-100 ML, Millipore Sigma, St. Louis, MO, United States) for 1 h at room temperature. Then, the sections were incubated with primary antibodies for 20 h at room temperature. The primary antibodies included mouse anti-C-terminal-binding protein-2 (CtBP2, 1:300, Cat # 612044; BD Biosciences, United States), mouse anti-glutamate receptor 2, clone 6C4 (GluA2, 1:200, Cat # MAB397; Millipore, Germany), rabbit anti-myosin 7a polyclonal (1:500, Cat # 25-6790; Proteus BioSciences, United States), and rabbit anti-nitrotyrosine polyclonal (1:300, Cat # 06-284; Millipore, Germany). CtBP2 and GluA2 antibodies were used together during the immuno-processing for assessing the disruption of ribbon synapses. The following day, the sections were washed in PBS and incubated overnight with the corresponding Alexa Fluor-conjugated secondary antibodies at room temperature. The secondary antibodies included goat anti-mouse Alexa Fluor 568 (IgG1, 1:500), goat anti-mouse Alexa Fluor 488 (IgG2a, 1:500), and Alexa Fluor647 goat anti-rabbit (1:500). Fluorescein-conjugated phalloidin (Cat # F432; Life Technologies, Carlsbad, CA, United States) was used to label F-actin. ProLong Gold antifade reagent containing 4′,6-diamidino-2-phenylindole (DAPI) nuclear stain (Cat # P36935; Invitrogen/Molecular Probes) was used to mount the stained specimens and the slides were kept overnight at 40°C.

### Image acquisition and analysis

Images were captured by using the Carl Zeiss Laser Scanning Systems (Zeiss LSM 780, Jena, Germany). The images of synaptic ribbons acquired using a 63X plan apochromatic objective with an effective numerical aperture of 1.4. Z-stacks of 20–25 slices (∼10 μm) were compressed, and 1,024 × 1,024 pixel size (8 bit depth) images were analyzed. At each image field, maximum intensity projection was generated from z-stack for counting the CtBP2 and GluA2 puncta. The area spanning 5 inner hair cells was defined using the freehand selection tool, and the number of puncta in background adjusted images (with a threshold value set to 45 out of 255) was counted using the “analyze particles” tool in the ImageJ/Fiji software (version IJ 1.46r). The images of hair cells and spiral ganglions were acquired using 40X plan apochromatic objective and 1.3 numerical aperture. Z-stacks of 10–15 slices (∼10 μm) were compressed, and 512 × 512 pixel size (8 bit depth) images were analyzed. To quantify the intensity of the immune staining, the pixel values were measured by using the ImageJ/Fiji software (version IJ 1.46r).

### Data analyses

Group size was determined statistically by using the data obtained in our previous studies. The G*Power 3.1.9.2 software was used to perform *a priori* power analysis to determine the sample size. Cohen’s measures were computed using the mean and standard deviation values obtained in our previous studies, and the actual power was calculated to be 0.95. Two-tailed unpaired *t*-test was used for statistical analysis and *p*-value of < 0.05 was considered significant. All results are expressed as mean ± standard deviation. Each replicate represents data derived from an individual animal.

## Results

### Four-week lead exposure increased the blood lead levels in mice

Blood lead levels were assessed by ICP-MS analysis immediately after the 28-day lead exposure. This analysis indicated that the exposure paradigm used in this study significantly increased the lead load (Figure 1B), which mimicked lead poisoning levels in most mammals (Dalefield, 2017). Nevertheless, the lead-exposed animals were generally healthy and maintained body weight similar to that of the controls.

### Outer hair cell activity was not affected by lead exposure

**FIGURE 2:**
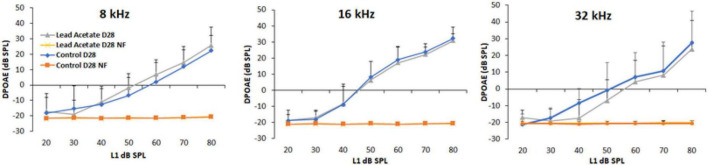
Effect of lead exposure on outer hair cell activity. Distortion product otoacoustic emissions (DPOAEs) were recorded in control and lead-exposed mice using 8, 16, and 32 kHz f2 stimuli. The traces labeled NF represent the noise floor of the DPOAE recordings. The distortion product amplitudes of mice with 28-day lead exposure were largely similar to that of unexposed controls. The results are expressed as mean ± standard deviation, *n* = 4–5.

DPOAE amplitudes were used to assess lead-induced changes in outer hair cell (OHC) activity. The otoacoustic emissions were recorded using 8, 16, and 32 kHz f2 stimuli for 1 day after 28-day lead exposure. Baseline DPOAEs were recorded in all animals prior to lead exposure and only animals with good baseline hearing were used in this study. A comparative analysis of the hearing thresholds recorded in the control and lead-exposed animals indicated that the distortion product amplitudes were largely similar in both groups of mice (Figure 2). Although C57BL6 mice have early onset of age-related hearing loss and the vulnerability of the hair cells to ototoxicants in 10-week-old mice is likely to be higher in this strain, these results suggest that lead exposure at this level does not affect the activity of the OHCs.

### Lead exposure did not cause hair cell loss

**FIGURE 3:**
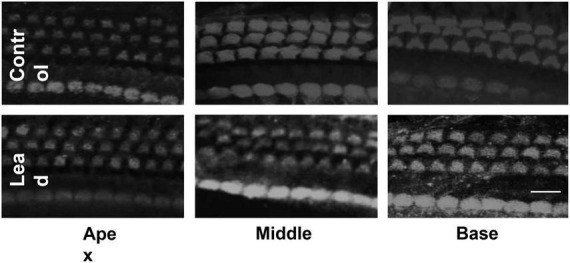
Effect of lead exposure on hair cell viability. Surface preparations of organ of Corti stained with anti-myosin 7a are illustrated. The outer and inner hair cells in the lead-exposed mice appear similar to that of controls and no hair cell loss was observed in all three turns of the organ of Corti. Images are representative of six replicates. Scale bar = 20 μm.

Outer and inner hair cells stained with anti-myosin 7a were examined *via* a confocal microscope (40 × magnification) to detect lead-induced hair cell loss. The confocal images indicated that lead exposure did not induce hair cell loss in the base, mid, and apical regions of the organ of Corti (Figure 3). The hair cells in the lead-exposed animals appeared similar to that of controls, suggesting that hair cells are not the primary targets in lead-induced ototoxicity.

### Cochlear ribbon synapses were disrupted by lead exposure

**FIGURE 4:**
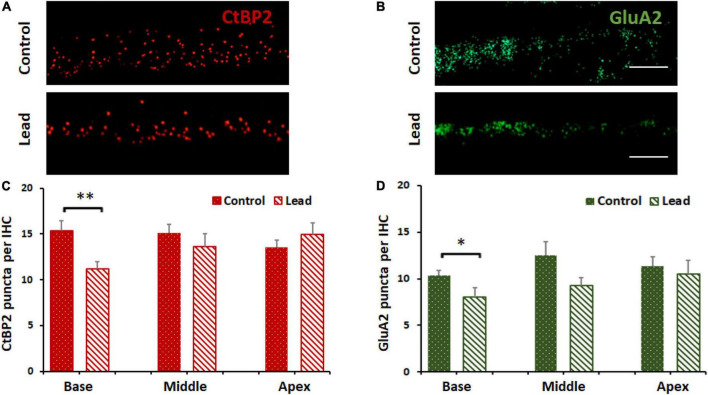
Lead-induced disruption of cochlear ribbon synapses. **(A)** Inner hair cells in the basal region of the organ of Corti probed with antibodies against C-terminal binding protein 2 (CtBP2, red stain) are illustrated. Lead exposure decreased the expression of this pre-synaptic marker in the inner hair cell synapses. Images are representative of 5–8 replicates, scale bar = 20 μm. **(B)** Inner hair cells in the basal region of the organ of Corti probed with antibodies against glutamate receptor subunits 2 (GluA2, green stain) are illustrated. Lead exposure decreased the expression of this post-synaptic marker in the inner hair cell synapses. Images are representative of 5–8 replicates, scale bar = 20 μm. **(C,D)** Counts of CtBP2 and GluA2 puncta indicated that lead exposure decreased the counts significantly in the basal region of the inner hair cells (IHC, *n* = 5–8). *p < 0.05, **p < 0.01. The results are expressed as mean ± standard error mean, *n* = 5–8.

Lead-induced loss of ribbon synapses was assessed by immunostaining the inner hair cells with anti-CtBP2 and anti-GluA2. The puncta representing the pre- and post-synaptic markers in the inner hair cells were counted *via* a confocal microscope (63 × magnification). The count of the stained puncta indicated that lead exposure significantly decreased the expression of CtBP2 and GluA2 in the inner hair cells located in the basal region of the organ of Corti (Figures 4A–D). This suggested that the ribbon synapses in the base of the cochlea were more susceptible to lead-induced disruption than those in the middle and apical regions.

### Lead exposure induced nitrative stress in the spiral ganglions

**FIGURE 5:**
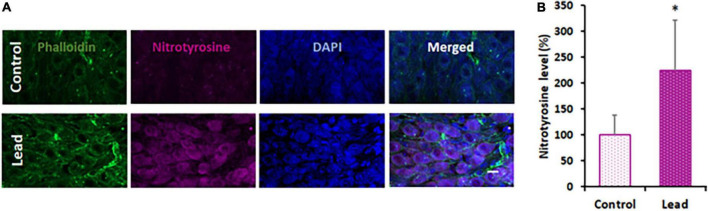
Lead-induced cochlear nitrative stress. **(A)** Spiral ganglions probed with anti-nitrotyrosine (magenta stain) indicated an increase in the level of nitrotyrosine after lead exposure. Phalloidin was used to stain actin (green) and DAPI was used to stain the nucleus (blue). Images are representative of 4–5 replicates, scale bar = 10 μm. **(B)** Quantification of nitrotyrosine staining indicated a significant increase in the spiral ganglion cells of lead-exposed mice. The results are expressed as mean ± standard deviation, *p < 0.05, *n* = 4–5.

Lead-induced cochlear nitrative stress was evaluated in the spiral ganglion cells located in the basal region of the organ of Corti by immunohistochemistry analysis with anti-nitrotyrosine. We focused on spiral ganglions in the basal region because lead exposure did not affect the ribbon synapses in the middle and apical regions. Moreover, we did not assess the nitrotyrosine levels in the hair cells because hair cells were not affected by lead exposure. The specificity of this immunoreaction was verified in our previous studies (Jamesdaniel et al., 2012). Nitrotyrosine levels were quantified by analyzing the intensity of the fluorescence of anti-nitrotyrosine using the ImageJ software. The fluorescence of DAPI was not quantified. The average intensity of five spiral ganglion cells per cochlear turn was calculated and normalized with corresponding actin intensities. The nitrotyrosine level in lead-exposed animals was significantly higher than that of the controls (Figures 5A,B). This suggested that lead exposure induced nitrative stress in the cochlea.

## Discussion

Environmental chemicals pose a significant health risk in urban neighborhoods because of historical contamination from anthropogenic sources, accumulation of multiple pollutants, and harmful interactions. Exposure to lead, one of the most commonly encountered environmental toxicants, is generally unavoidable because it is ubiquitous in air, water, and soil. Residents of inner cities with older infrastructure are exposed to higher levels of lead because most of the houses contain lead paints and/or lead pipes. Hearing impairment, the third most common chronic physical condition (Blackwell et al., 2014) reported by 15% of Americans between the ages of 20 and 69 years (Hoffman et al., 2006), is associated with long-term exposure to lead (Shargorodsky et al., 2011; Choi et al., 2012; Ghiasvand et al., 2016; Rosati and Jamesdaniel, 2020). Since the United Nations projects that 68% of the world population will live in urban areas by 2050, delineating the otopathology associated with lead exposure is important for discovering effective strategies to protect vulnerable communities from lead-induced hearing impairment. This study provides the first evidence indicating lead exposure, even at levels that do not damage OHCs, and disrupts the cochlear ribbon synapses. In addition, this study suggests that nitrative stress may play a role in contributing to this otopathology.

Outer hair cells, the sensory receptor cells in the cochlea, are highly susceptible to damage induced by agents such as noise and ototoxic drugs. In previous studies, lead-induced hearing impairment in rodents was detected by recording auditory brainstem responses (ABRs; Liu et al., 2013; Jamesdaniel et al., 2018). However, the ABRs can reflect ototoxin-induced damage to different regions of the auditory pathway. Therefore, the threshold shifts observed in the ABRs do not always indicate OHC-specific damage. To verify whether lead exposure induces OHC damage, this study employed an exposure paradigm that increased the blood lead levels to ∼32 μg/dl, which mimics lead poisoning levels in most mammals (Dalefield, 2017), and then assessed the DPOAEs, which reflect OHC activity, and OHC loss. Exposure at this level to young-adult mice did not affect the structure and function of the OHCs as reflected by the otoacoustic emissions and immunohistochemistry analysis. Although we did not measure DPOAEs at frequencies higher than 32 kHz, which represents the mid-basal region, the morphological examination indicated that lead exposure did not affect the hair cells in the basal region. These results are consistent with the findings of other studies (Carlson and Neitzel, 2018) and suggest that the OHCs are not the primary targets in lead-induced ototoxicity.

Although OHCs were not damaged, wave 1 latencies were prolonged in ABRs recorded post lead exposure (Liu et al., 2011; Zhang et al., 2019), suggesting interference with the transmission of auditory signals potentially due to cochlear neuropathy. Several studies have reported that lead-induced neuronal damage is facilitated by glutamate excitotoxicity (Lasley and Gilbert, 2002), excessive Ca_2_^+^ influx (Ferguson et al., 2000; Kern et al., 2000), and oxidative stress (Ding et al., 2000; Patrick, 2006; Antonio-Garcia and Masso-Gonzalez, 2008), which are mechanisms attributed to cochlear synaptopathy and neurodegeneration of the auditory nerve (Liberman and Kujawa, 2017; Sebe et al., 2017). To verify whether lead exposure induces cochlear synaptopathy, pre- and post-synaptic biomarkers of cochlear ribbon synapses were evaluated. Immunohistochemistry analysis indicated that the number of CtBP2 and GluA2 puncta in the ribbon synapses were significantly reduced in the inner hair cells located in the basal region of the organ of Corti after lead exposure. These results suggest that even exposure at levels that do not affect OHC function can disrupt the cochlear ribbon synapses.

The mechanism underlying lead-induced disruption of cochlear ribbon synapses is unknown. Damage to the cochlear ribbon synapses is generally attributed to glutamate excitotoxicity (Liberman and Kujawa, 2017) and excessive Ca^2+^ influx into afferent terminals (Sebe et al., 2017), which can eventually trigger a cascade of reactions resulting in oxidative stress (Maher et al., 2018). As lead-induced neuronal damage is facilitated by glutamate excitotoxicity (Lasley and Gilbert, 2002), excessive Ca2 + influx (Ferguson et al., 2000; Kern et al., 2000), and oxidative stress (Ding et al., 2000; Patrick, 2006; Antonio-Garcia and Masso-Gonzalez, 2008), lead-induced disruption of cochlear ribbon synapses could be mediated by any of these mechanisms. Particularly, redox mechanisms can facilitate cochlear synaptopathy because many synaptic proteins are highly susceptible to nitration (Bradley and Steinert, 2016). Moreover, nitration of synaptic proteins such as SNAP-25, Munc-18, Synaptophysin, Dynamin, and Synaptotagmin, has been reported in other models (Di Stasi et al., 2002; Vrljic et al., 2011; Mallozzi et al., 2013). In a previous study, lead exposure induced oxidative stress, a precursor of nitrative stress, in the cochlea (Jamesdaniel et al., 2018). The reaction of ROS (e.g., O_2_^⋅–^) with reactive nitrogen species (e.g., NO) leads to the formation of peroxynitrite (ONOO^–^), which can react with susceptible proteins to form nitrotyrosine, an indicator of nitrative damage to proteins. To verify whether lead exposure induces cochlear nitrative stress, changes in nitrotyrosine levels were evaluated. Immunohistochemistry analysis indicated that the nitrotyrosine levels in the spiral ganglions were significantly increased after lead exposure. As lead exposure induces cochlear nitrative stress, the synaptic proteins in the cochlear ribbon synapses are potential targets for nitration, which could eventually contribute to the disruption of ribbon synapses observed after lead exposure.

## Conclusion

This study provides the first evidence for lead-induced disruption of cochlear ribbon synapses and suggests that nitrative stress may play a role in contributing to this otopathology. One of the limitations of this study is the lack of functional data to corroborate lead-induced cochlear synaptic disruption. Nevertheless, the findings of this study are important because they suggest that even exposure at levels that do not damage OHCs can induce cochlear synaptopathy, which in turn can affect the quality of life and productivity. Follow-up studies on cochlear synaptic proteins that are nitrated by lead exposure would provide important insights into the nitrative stress mechanism underlying lead-induced cochlear synaptopathy and identify potential targets for intervention.

## Data availability statement

The original contributions presented in this study are included in the article/supplementary material, further inquiries can be directed to the corresponding author.

## Ethics statement

The animal study was reviewed and approved by Institutional Animal Care and Use Committee, Wayne State University.

## Author contributions

SJ contributed to the study concept and design. RR and JB performed research. SJ, RR, and JW analyzed the data. SJ and RR wrote the manuscript. All authors contributed to the article and approved the submitted version.
